# Stool Parasitological Examination: A Diagnostic Methodology Under Threat of Extinction

**DOI:** 10.1590/0037-8682-0333-2025

**Published:** 2026-02-09

**Authors:** Maria Fantinatti, Sidnei da Silva, Claudia Maria Antunes Uchôa, Alda Maria Da-Cruz

**Affiliations:** 1 Disciplina de Parasitologia, Faculdade de Ciências Médicas, Universidade do Estado do Rio de Janeiro, Rio de Janeiro, RJ, Brasil.; 2 Laboratório de Parasitologia, Instituto Nacional de Infectologia, Fundação Oswaldo Cruz, Rio de Janeiro, RJ, Brasil.; 3 Disciplina de Parasitologia, Departamento de Microbiologia e Parasitologia, Instituto Biomédico, Universidade Federal Fluminense, Niterói, RJ, Brasil.; 4 Laboratório Interdisciplinar de Pesquisas Médicas, Instituto Oswaldo Cruz, Fundação Oswaldo Cruz, Rio de Janeiro, RJ, Brasil.

**Dear Editor:**

Intestinal parasitic infections comprise diseases caused by protozoa (unicellular protists, based on phylogenetic reclassifications) and helminths that remain major public health concerns due to their high prevalence and morbidity. These infections often progress silently, complicating diagnosis and treatment. In Brazil, as in many other countries, the main diagnostic method for these parasitic diseases is the stool parasitological examination (SPE)^1^. SPE includes several techniques designed according to the biological characteristics of the etiological agent under investigation. No single technique can detect all intestinal parasites; therefore, combined techniques are recommended, especially where the circulating parasitic fauna is unknown^2^ ^,^ ^3^. It is important to choose the appropriate technique in advance based on the etiological hypothesis, to improve diagnostic accuracy.

The first known SPE was likely performed by Antony van Leeuwenhoek, one of the inventors of the microscope, in 1681, when he examined his own diarrheal stool using his newly developed instrument. The technique he employed-now called direct examination-remains in use today, particularly for diseases such as balantidiasis, in which the use of direct examination associated with the Lutz technique was more effective^4^.

Although molecular and immunological diagnostic tools now offer greater sensitivity, they typically detect a limited range of parasites and remain costly. Moreover, their relatively high cost, particularly in developing countries, where infection rates are highest and investment in research is limited-continues to hinder their implementation in routine clinical practice^5^. Since the early 20th century, SPE has remained the standard diagnostic method for intestinal parasites because it detects various developmental stages of helminths (eggs, larvae, and occasionally adult worms) and protozoa (trophozoites and (oo)cysts) at low cost^1^ ^,^ ^6^.

Beyond estimating the prevalence of parasitic diseases, SPE supports appropriate therapeutic decisions and treatment evaluation, thereby reducing the duration of host-parasite interaction and its consequences. However, many healthcare professionals undervalue SPE due to its perceived low sensitivity. Although its sensitivity decreases with low parasite burden, SPE remains highly manual, with few methodological advances since the 1950s to 1980s, despite major scientific progress.

In addition to the inherent limitations of the method, false-negative results may stem from (1) low parasite burden and intermittent shedding; (2) bad samples coditions; (3) inadequate microscopist training; and (4) improper test requisition^7^ ^-^ ^9^. Except for parasite burden, these limitations can be minimized through health education, technical training, and effective communication. Thus, SPE accuracy depends on preanalytical factors, including clinical suspicion informed by patient history, proper sample collection and storage, recommended sample number, appropriate SPE technique selection or combination, use of staining procedures, and trained personnel^1^ ^,^ ^10^.

Recently, the diagnosis of intestinal parasitic infections has increasingly been replaced by empirical treatment. This approach is especially common when patients experience discomfort or stigma regarding stool sample submission. However, this approach often leads to inappropriate drug selection, incorrect dosing, or unnecessary treatment. The normalization of parasitic infections within affected populations, combined with the indiscriminate and routine use of antiparasitic drugs-typically administered every 6-12 months and mistakenly regarded as a prophylactic measure-may contribute to the emergence of drug-resistant strains^11^.

This issue is concerning given the limited therapeutic options for these infections. Furthermore, feces contain diverse microorganisms, most of which are non-pathogenic and essential for a healthy microbiome. Thus, treatment of non-pathogenic parasites or those with uncertain pathogenicity should be carefully evaluated to prevent unnecessary drug use and its potential consequences^12^.

This gradual decline in the demand for parasitological diagnosis has diminished motivation among laboratory professionals specializing in SPE and reduced investment in training and professional development. These specialists often lack technical support and receive minimal financial compensation, which is often reimbursed at a negligible rate (approximately $0.12 USD per test in Brazil)^10^.

Although automated diagnostic tools and real-time multiplex PCR and mehtods based on artificial inteligence approachs healthcare systems remains limited or confined to research. None of the newer techniques can yet fully replace microscopy, underscoring the need for standardized guidelines that integrate advanced technologies with microscopy rather than replacing it. With appropriate implementation, microscopy will continue to play a central role in intestinal parasitology, allowing simultaneous detection of a wide range of parasites within a short period^5^. 

Therefore, research investments are urgently needed to develop more sensitive and affordable diagnostic methodologies. However, until such innovations emerge, SPE remains indispensable for accurately diagnosing intestinal parasites, identifying specific parasite species, in some cases, and supporting the selection of appropriate treatment. Strengthening health education, professional training, and communication initiatives is essential to highlight the value of this methodology, foster specialized expertise, and enhance diagnostic requisitions, patient guidance, and laboratory practices.
